# Perioperative platelet count in peripheral blood is associated with the early stage of PND after major orthopedic surgery: a prospective observational study

**DOI:** 10.1186/s12877-022-02899-7

**Published:** 2022-03-14

**Authors:** Ruiqun Wang, Rui Gao, Xiaoyu Xie, Hai Chen, Qi Zhao, Xueying Zhang, Changteng Zhang, Liyun Deng, Peilin Lv, Qin Zheng, Tao Zhu, Chan Chen

**Affiliations:** 1 Department of Anesthesiology, West China Hospital, Sichuan University, Chengdu, 610041 Sichuan China; 2 Laboratory of Anesthesia and Critical Care Medicine, National-Local Joint Engineering Research Centre of Translational Medicine of Anesthesiology, West China Hospital, Sichuan University, Chengdu, 610041 Sichuan China; 3 Department of Respiratory and Critical Care Medicine, Targeted Tracer Research and Development Laboratory, West China Hospital, Sichuan University, Chengdu, Sichuan China; 4 Department of Laboratory Medicine, West China Hospital, Sichuan University, Chengdu, Sichuan China

**Keywords:** Perioperative neurocognitive disorders, Perioperative platelet count, Biomarker, Elderly major orthopedic surgery

## Abstract

**Background:**

Perioperative neurocognitive disorders (PND) are common complications of major surgery among elderly patients, remarkably decreasing patients’ life quality. Platelet count has been proved to be an essential factor in inflammation. However, as far as we know, the relationship between platelet count and PND is not clear yet in the orthopedic area. PND could be a long-term disease, which sometimes lasts for several years, and it is meaningful to find a biomarker of PND at the early stage. Thus, we designed this study to find out the association between perioperative platelet count and occurrence of PND, and determine whether preoperative platelet count could be a biomarker of the early stage of PND.

**Methods:**

A prospective observational study was performed on the patients who would take total knee arthroplasty or total hip arthroplasty. Their peripheral platelets were counted by blood routine examination 1 day before and 3 days after the surgery. And we assessed their neurocognitive functions 1 day before and 3 days after the surgery. These data were recorded and analyzed to find out the relationship between platelet count and the occurrence of PND.

**Results:**

Eventually, 70 patients finished the whole process, and 14 of them developed PND. The median preoperative platelet count in the PND group was significantly higher than that in the non-PND group (239 vs 168 × 10^9/L, *p* = 0.009). Preoperative platelet count was an independent risk factor for PND (odds ratio = 1.014, 95% confidence interval [CI] 1.000–1.027, *P* = 0.043) in the logistic multivariable regression, while the area under the curve of the receiver operating characteristic curve of the prediction model was 0.796 (95% CI 0.676–0.916).

**Conclusions:**

The higher preoperative and postoperative level of platelet count in the peripheral blood were associated with the early stage of PND, and preoperative platelet count could be a potential predictor of the early stage of PND in patients undergoing major orthopedic surgeries.

**Trial registration:**

Chinese Clinical Trial Registry: ChiCTR2000033001, registration date: 17 May 2020.

**Supplementary Information:**

The online version contains supplementary material available at 10.1186/s12877-022-02899-7.

## Background

Perioperative neurocognitive disorders (PND), which were brought up in 2018, are common complications of major surgery among elderly patients, including cognitive impairment diagnosed before operation and postoperative acute and chronic cognitive dysfunction that could be diagnosed from 1 day to 1 year after surgery [1]. This concept combines the postoperative delirium (POD) and postoperative cognitive dysfunction (POCD). At the same time, POD is defined as an acute disorder of attention and cognition after surgery, and POCD used to be a concept but not a clinical diagnosis, representing a significant decline in neurocognitive performance in attention, orientation, memory, verbal fluency, coordination and so on [2, 3]. PND has been proved to increase the cost of care, reduce the quality of life, increase the risk of long-term cognitive decline and mortality [4–9]. Furthermore, the relationship between PND and cardiac surgery has been discussed widely. In contrast, in non-cardiac surgery, the incidence of PND ranges between 8.9 and 46.1%, depending on the study and type of surgery [10]. As the PND may have a long course and may even develop to Alzheimer‘s disease (AD) [11], which is hard to be cured by most clinical drugs [12], it is essential to diagnose and treat it at the early stage. Meanwhile, many recent studies focused on mechanisms and biomarkers at the early stage of PND, such as on the third day after surgery [13–15]. However, platelet count has not been reported as a biomarker in the early stage of PND.

Among many non-cardiac surgeries, total knee arthroplasty (TKA) and total hip arthroplasty (THA) are prevalent types of major orthopedic surgery. As the proportion of older people increases in China, the prevalence of osteoarthritis on the knee has risen to 21.85% [16], becoming a heavy burden for society. TKA is one of the most helpful solutions to knee osteoarthritis [17], while THA has a similar circumstance to TKA [18, 19]. Recent studies have shown that THA and TKA could induce an obvious inflammation reaction process via cell-immune response, genomic storm, or postoperative infection [20–22]. Moreover, several studies have proved that TKA and THA are risky for venous thromboembolism [23, 24]. Besides being the main reason for thrombosis, platelets have been an essential factor in inflammation by transferring signals in immune cell-cell interactions, activating and recruiting leukocytes to the inflammation site. Even its secreting exosomes can promote inflammation [25–27]. PND has been reported to result from a central neuroinflammatory response to surgery [28, 29]. However, as far as we know, the relationship between platelet count and occurrence of PND has never been investigated yet in the orthopedic area.

In this study, we designed this prospective observational study to determine whether perioperative platelet count would be a biomarker of the early stage of PND. Besides, a predicting model for the occurrence of PND was also constructed among elderly patients undergoing major orthopedic surgery.

## Methods

### Patients

The patients who took TKA or THA in West China Hospital between June 2020 and May 2021 were included in the study. The inclusion criteria were: (1) patients older than 55 years; (2) requiring a TKA or a THA surgery; The exclusion criteria were: (1) history of mental diseases; (2) patients with current Neurological disease; (3) left ventricle ejection fraction < 40%; (4) patients who refused or were unable to finish the neurocognitive evaluation; (5) preoperative Mini-Mental Stare Examination (MMSE) less than required score (illiteracy < 17; primary school < 20; middle school and higher < 24).

### Clinical data collection

Common demographic factors were collected when the patients were admitted to the hospital, for example, age, gender, body mass index (BMI), and education level. Moreover, professional doctors performed a cardiac examination for the New York Heart Association classification (NYHA). The hemocyte indexes, hepatic and renal functions were assessed by blood routine examination 1 day before and 3 days after the surgery. Perioperative change of platelet was defined as postoperative platelets minus preoperative platelets.

### Blood collection

A well-trained nurse collected the blood samples 1 day before and 3 days after the surgery, and the blood analysis was performed by the hematology analyzer (SYSMEXXN-10, Sysmex, Japan) in the clinical lab. Therefore, we mainly focused on the platelet count before and after the surgery.

### Neurocognitive evaluation

To evaluate the patients’ neurocognitive levels, a well-trained investigator performed a questionnaire to roundly assess their neurocognitive functions 1 day before and 3 days after the surgery. The questionnaire included: (1) Mini-Mental State Examination (MMSE); (2) Word Immediate Recall Test, testing ability of short-memory; (3) Image Immediate Recall Test, testing ability of short-term visual memory; (4) Trail Making Test A, testing hand-eye coordination; (5) Digit Span Test, testing concentration as well as attention; (6) Digit Symbol Coding Test, testing psychomotor speed; (7) Word Delayed Recall Test, testing ability of long-memory; (8) Word Delayed Interference Test; (9) Image Delayed Recall Test, testing ability of delay recall; (10) Image Delayed Interference Test and (11) Verbal Fluency Test, testing fluency and executive function. At the very beginning, patients with severe cognitive dysfunction were excluded by the lower MMSE score than we required. Each qualified patient gets scores of these tests preoperatively and postoperatively. We collected all of these scores and then obtained the standard deviation (SD) of every preoperative test. We defined a worse test performance as a negative changed score, with an absolute value larger than 1.5 times SD. Patients with no less than two negative changed scores, except for MMSE, were diagnosed as PND [30, 31]. According to this standard, we divided the participants into the PND group and the non-PND group. Finally, we defined two decreased scores as mild PND and more than two decreased scores as severe PND.

### Anesthesia and surgery

All patients got the same preoperative preparation, including ECG monitoring, blood oxygen saturation monitoring, arterial blood pressure, and central venous pressure monitoring. As for the anesthesia method, we took combined anesthesia of inhalation and intravenous. The main anesthetic included sevoflurane, desflurane, propofol, cisatracurium besylate, and lidocaine for topical anesthesia. During the surgery, the patients were given mechanical ventilation at 12 times/min, with a tidal volume of 6–8 ml/kg. After the surgery, patients were treated with a drainage tube, which was placed locally to reduce the hematoma and relieve pain. And antibiotics were administered on the day before and on the day of surgery to prevent infection, which could be a catastrophic complication of TKA and THA. The second day after surgery, low molecular weight heparin was injected to avoid a deep venous thrombosis. Early rehabilitation training was encouraged.

### Statistics analysis

All of the continuous variables were shown as median with interquartile range (IQR). And all of the categorical variables were shown as a number with a percentage. We used the Mann-Whitney U test for comparisons of continuous variables between different groups and the Fisher exact test for comparisons of categorical variables. The receiver operating characteristic curve (ROC curve) was used to determine the most appropriate threshold of platelet count decided by the Youden Index, and the area under the curve (AUC), 95% confident interval (95%CI), as well as specificity (spe) and sensitivity (sen) were displayed. Furthermore, multivariate logistic regression analysis, which included potential risk factors, was performed to determine the independent risk factors of the early stage of PND. Results were shown as odds ratio (OR) and 95%CI. As a result, we also built a prediction model for the occurrence of PND and performed the ROC curve to evaluate the prediction effect of the model. We took education, gender, duration of surgery, and preoperative platelet into consideration. Among them, the preoperative platelet and duration of surgery were analyzed as continuous variables. On the contrary, education was divided into two groups by the middle school; gender was divided into two groups as well. All statistical analyses were performed by the IBM SPSS Statistical 26. We regarded a two-sided *P* < 0.05 as statistically significant.

## Results

### Patients

**Fig. 1:**
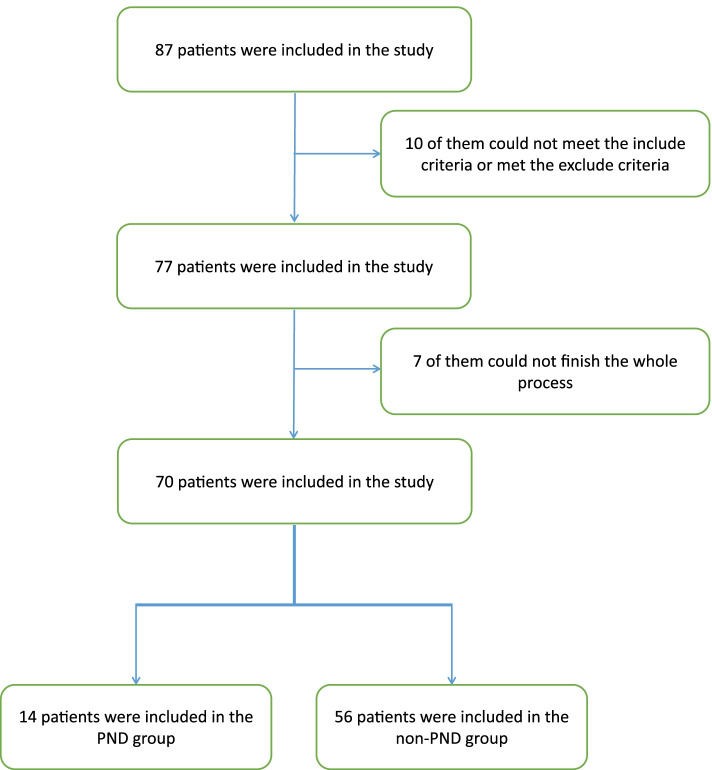
Flow diagram of participants. The figure showed the whole process of patients’ inclusion and exclusion

Totally 87 patients were enrolled in the study, and 70 of them finished the whole process (Fig. 1). According to the neurocognitive evaluation, 14 were classified into the PND group, while the others were assigned to the non-PND group. All the baseline information and laboratory examinations of the PND and non-PND groups were shown in Tables 1 and 2. We observed that women developed significantly more PND than men (Table 1). In addition, no significant cognitive difference existed between these two groups before the surgery, suggesting that all of the patients were in a similar cognitive condition at the beginning (Table 3). However, after surgery, the PND group performed significantly worse than the non-PND group in Word Immediate Recall Test (*P* = 0.009), word delayed recall test (*P* = 0.001), word delayed interference test (*P* = 0.001), and Image Delayed Recall Test (*P* = 0.028) (Table 4). Meanwhile, we did not observe a significant difference between the mild PND group and severe PND group in their baseline information and clinical examinations (Supplementary Files 1 and 2).

**Table 1:** Preoperative variables of the total cohort, PND and non-PND group

Preoperative variables	Total cohort *N* = 70	PND group *N* = 14	Non-PND group *N* = 56	*P*
Age (y)	67.0 (62.3–73.0)	69.5 (64.3–73.5)	67.0 (62.8–73.0)	0.670
BMI (kg/m^2)^	24.9 (22.0–27.3)	24.9 (21.7–28.1)	25.0 (22.2–27.2)	0.831
Gender				0.026*
Male	23 (32.9%)	1 (4.3%)	22 (95.7%)	
Female	47 (67.1%)	13 (27.7%)	34 (72.3%)	
Education				0.760
No lower than middle school	45 (64.3%)	8 (17.8%)	37 (82.2%)	
Lower than middle school	25 (35.7%)	6 (24.0%)	19 (76.0%)	
Operation type				0.148
TKA	37 (52.9%)	5 (13.5%)	32 (86.5%)	
THA	33 (47.1%)	9 (27.3%)	23 (72.7%)	
NYHA classification				1.000
≥ ΙΙ	23 (32.9%)	4 (17.4%)	19 (82.6%)	
< ΙΙ	47 (67.1%)	10 (21.3%)	37 (78.7%)	
Hypertension				0.744
Yes	19 (27.1%)	3 (15.8%)	16 (84.2%)	
No	51 (72.9%)	11 (21.6%)	40 (78.4%)	
If atherosclerosis in lower limbs				1.000
Yes	30 (42.9%)	6 (20.0%)	24 (80.0%)	
No	40 (57.1%)	8 (20.0%)	32 (80.0%)	
Hemoglobin (g/L)	136 (127–143)	135 (125–145)	136 (127–143)	0.848
Hematokrit	0.42 (0.39–0.44)	0.42(0.38–0.45)	0.42(0.39–0.44)	0.918
Platelet (10^9/L)	184 (151–232)	239(191–255)	168(151–215)	0.009**
Leukocyte (10^9/L)	5.78 (4.85–6.64)	5.85(4.72–6.81)	5.76(4.85–6.71)	0.901
Neutrophil percentage (%)	62.5 (55.4–69.3)	60.9(54.7–67.4)	63.0(55.5–70.1)	0.528
Neutrophil count (10^9/L)	3.57 (3.04–4.25)	3.72(3.09–4.25)	3.53(2.98–4.32)	0.947
Lymphocyte (10^9/L)	1.52 (1.19–1.96)	1.70(1.10–2.21)	1.52(1.22–1.96)	0.676
PLR	118.3 (83.6–170.0)	136.5(91.3–210.1)	117.0(83.6–161.3)	0.311
Monocyte (10^9/L)	0.39 (0.33–0.48)	0.37(0.35–0.49)	0.39(0.32–0.48)	0.581
ALT (U/L)	17.0 (12.8–23.3)	15.5(13.5–31.5)	18(12.3–22.8)	0.982
AST (U/L)	21.5 (19.9–26.0)	20.5(18.0–30.0)	22(19–26)	0.895
ALB (g/L)	43.9 (41.6–46.2)	44.2(41.3–46.3)	43.9(42.0–46.2)	0.769
TBil (μmol/L)	10.2 (8.0–13.4)	10.6(8.8–14.1)	10(8–13.5)	0.725
HDL (mmol/L)	1.4 (1.1–1.7)	1.5(1.2–1.7)	1.4(1.1–1.6)	0.29
LDL (mmol/L)	3.2 (2.3–4.1)	3.4(2.6–4.1)	3.2(2.3–4.1)	0.803
Serum creatinine (μmol/L)	66.0 (56.8–78.0)	63.5(55.5–71.8)	66.0 (57.0–79.8)	0.287
Blood glucose (mmol/L)	5.5 (5.1–6.2)	5.6(4.8–6.0)	5.5(5.2–6.3)	0.681
PT (s)	10.5 (10.1–10.8)	10.5(9.9–10.7)	10.6(10.1–10.8)	0.423
APTT (s)	26.2 (25.0–27.1)	25.7(24.7–26.7)	26.2(25.0–27.1)	0.366
INR	0.97 (0.92–1.00)	0.97(0.91–0.98)	0.96(0.93–1.00)	0.591
Duration of surgery (min)	64.5 (54.5–85.0)	61.5(54.3–95.8)	65.0 (53.5–83.8)	0.994
Duration of anesthesia (min)	113.0 (100.0–144.5)	109.0 (99.8–153.5)	114.5(100.3–142.3)	0.78

Data are presented as median with IQR for continuous variables and as number for categorical variables. The *P*-value is calculated by the Mann-Whitney U test for continuous variables and by Fisher’s exact test for categorical variables

*PND* Perioperative neurocognitive disorders, *BMI* Body mass index, *TKA* Total knee arthroplasty, *THA* Total hip arthroplasty, *NYHA* New York Heart Association, *PLR* Platelet-to-lymphocyte ratio, *ALT* Alanine aminotransferase, *AST* Aspartate aminotransferase, *ALB* Albumin, *TBil* Total bilirubin, *HDL* High density lipoprotein, *LDL* Low density lipoprotein, *PT* Prothrombin time, *APTT* Activated partial thromboplastin time, *INR* International normalized ratio

^*^P means *P*-value < 0.05

^**^P means *P*-value < 0.01. We diagnose the systolic pressure ≥ 140 mmHg and/or diastolic pressure ≥ 90 mmHg preoperative as hypertension

**Table 2:** Postoperative variables of the total cohort, PND and non-PND group

Postoperative variables	Total cohort *N* = 70	PND group *N* = 14	Non-PND group *N* = 56	*P*
Hemoglobin	118 (110–126)	118(110–128)	118(110–125.5)	0.769
Hematokrit	0.36 (0.34–0.38)	0.36(0.33–0.41)	0.37(0.34–0.38)	0.947
Platelet (10^9/L)	164 (139–208)	208(188–221)	156(137–194)	0.003**
Perioperative Change of platelet	−17.5 (−37.0–0.5)	−25.5 (−38.3- -8.5)	−14.5 (−28.8–1.5)	0.290
Leukocyte (10^9/L)	10.54 (8.91–13.62)	11.66(9.33–13.67)	10.41(8.86–13.80)	0.849
Neutrophil percentage (%)	85.1 (81.1–87.9)	84.1(82.9–87.3)	85.2(80.7–88.0)	0.763
Neutrophil count (10^9/L)	9.09 (7.26–11.78)	9.81(8.05–11.47)	8.86(7.21–11.80)	0.775
Lymphocyte (10^9/L)	1.04 (0.77–1.33)	1.2(0.77–1.45)	1.00(0.76–1.29)	0.374
PLR	170.9 (113.5–230.2)	184.6(128.6–251.3)	165.7(111.0–225.3)	0.284
Change of PLR	39.3 (2.0–83.7)	51.6(−1.1–89.2)	38.2(3.2–73.6)	0.725
Monocyte (10^9/L)	0.70 (0.45–0.88)	0.73(0.32–0.79)	0.68(0.45–0.89)	0.638
ALT (U/L)	16.0 (13.0–20.3)	17.5(14–29.5)	15.5(12.3–19)	0.245
AST (U/L)	21.5 (18.8–27.3)	24.5(19.8–34.3)	21(18–26)	0.112
ALB (g/L)	37.9 (35.8–39.7)	37.2(36.1–40.9)	38.1(35.7–39.6)	0.953
TBil (μmol/L)	11.4 (9.5–15.6)	11.1(8.9–14.1)	11.6(9.9–15.7)	0.454
HDL (mmol/L)	1.3 (1.1–1.6)	1.5(1.3–1.7)	1.3(1.1–1.6)	0.207
LDL (mmol/L)	2.7 (2.2–3.6)	2.7(2.1–3.5)	2.7(2.2–3.6)	0.843
Serum creatinine (μmol/L)	67.0 (58.8–80.0)	63(58–71.8)	67(58.3–80.8)	0.287
Blood glucose (mmol/L)	7.1 (6.2–8.1)	7.1(6.0–7.3)	7.2(6.2–8.3)	0.287

Data are presented as median with IQR for continuous variables and as the number for categorical variables. The *P*-value is calculated by the Mann-Whitney U test for continuous variables and by Fisher’s exact test for categorical variables

*PND* Perioperative neurocognitive disorders, *PLR* Platelet-to-lymphocyte ratio, *ALT* Alanine aminotransferase, *AST* Aspartate aminotransferase, *ALB* Albumin, *TBil* Total bilirubin, *HDL* High density lipoprotein, *LDL* Low density lipoprotein

^*^P means *P*-value < 0.05

^**^P means P-value < 0.01

**Table 3:** Difference of preoperative cognitive function

Tests	Total cohort	PND group	Non-PND group	*P*
MMSE	26(25–28)	26(25–29)	27(25–28)	0.853
Word Immediate Recall Test	13(11–16)	13.5(10–17)	13(11–16)	0.848
Image Immediate Recall Test	9(7–11)	9.5(9–11)	9(7–11)	0.404
Trail Making Test A	100.5(71–144.5)	108.5(69–159)	100.5(69–136)	0.809
Digit Span Test	18(16–21)	18.5(16–21)	18(16–21.5)	0.735
Digit Symbol Coding Test	25(19–32)	25(18.5–34)	25(19–32)	0.808
Word Delayed Recall Test	4(2–6)	4(2.5–6)	4(2–5.5)	0.377
Word Delayed Interference Test	22(20–23)	21.5(19.5–23)	22(21–23)	0.666
Image Delayed Recall Test	3(2–4)	3(2–4)	3(2–4)	0.493
Verbal Fluency Test	33(28–38)	29(26–35)	35(29–38.5)	0.126

Data are presented as median with IQR. The *P*-value is calculated by the Mann-Whitney U test

*PND* Perioperative neurocognitive disorders

^*^P means *P*-value < 0.05

^**^P means *P*-value < 0.01

**Table 4:** Difference of postoperative cognitive function

Tests	Total cohort	PND group	Non-PND group	*P*
Word Immediate Recall Test	14(11–18)	11.5(7.5–14)	15.5(11–19)	0.009**
Image Immediate Recall Test	9.5(6–12)	8.5(5–12)	10(6–12)	0.338
Trail Making Test A	104.5(66–145)	111(64.5–177.5)	104.5(65–139.5)	0.607
Digit Span Test	18(14–20)	14.5(12.5–20)	18(15–21)	0.103
Digit Symbol Coding Test	24.5(18.5–31)	21.5(16.5–28)	25(20–31.5)	0.338
Word Delayed Recall Test	3(1.5–6)	2(0–2)	4(3–6.5)	0.001**
Word Delayed Interference Test	21(18–23)	18(15.5–19.5)	22(20–23)	0.001**
Image Delayed Recall Test	3(2–5)	2(0.5–3)	3(2–5)	0.028*
Verbal Fluency Test	33(28–38)	29(22.5–36.5)	33.5(28–39)	0.179

Data are presented as median with IQR. The *P*-value is calculated by the Mann-Whitney U test

*PND* Perioperative neurocognitive disorders

^*^P means *P*-value < 0.05

^**^P means *P*-value < 0.01

### Relationship between preoperative platelet count and occurrence of PND

**Fig. 2:**
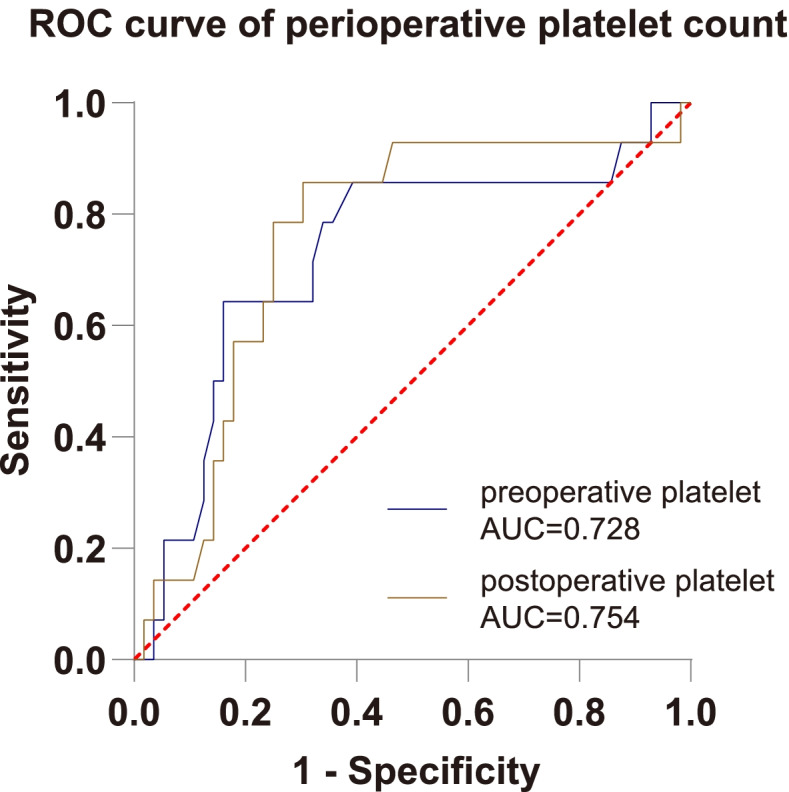
Receiver-operating characteristic (ROC) curves for preoperative platelet and postoperative platelet. The figure showed the ROC curves for preoperative platelet and postoperative platelet, whose area under the curve (AUC) were 0.728 and 0.754 respectively

**Fig. 3:**
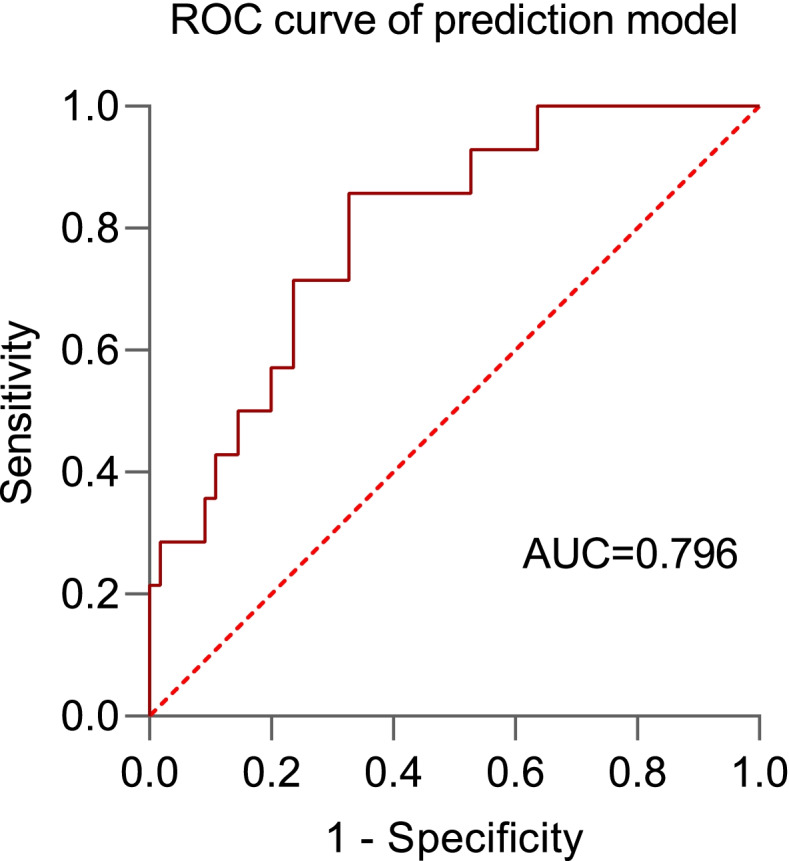
Receiver-operating characteristic (ROC) curve for prediction model of occurrence of PND. The figure showed the ROC curves for the prediction model of occurrence of PND, whose area under the curve (AUC) was 0.796

The preoperative platelet count level in the PND group was significantly higher than that in the non-PND group (*P* = 0.009, Table 1). Then, according to the ROC curve, the cut-off value (229, spe:0.839, sen:0.643) of preoperative platelet count was determined by Youden Index, and the AUC was 0.728 (*P* = 0.009, 95%CI 0.569–0.888, Fig. 2). To ensure whether preoperative platelet count was an independent risk factor of occurrence of PND, a multivariable logistic regression analysis was performed. The results showed that the *P*-value of the preoperative platelet count was 0.043 and OR was 1.014 (95%CI 1.000–1.027), meaning that it was an independent risk factor of occurrence of PND (Table 5). Finally, the prediction model of PND was shown, $P=\frac{e^{1.851\ (Gender)+0.729\ (Education)+0.014\ \left( Preoperative\ platelet\ count\right)+0.009\ \left( Duration\ of\ surgery\right)+\left(-6.457\right)}}{1+{e}^{1.851\ (Gender)+0.729\ (Education)+0.014\ \left( Preoperative\ platelet\ count\right)+0.009\ \left( Duration\ of\ surgery\right)+\left(-6.457\right)}}$ and the model’s predictive effect was examined by the ROC curve (Fig. 3), whose AUC was 0.796 (*P* = 0.001, 95%CI 0.676–0.916). The results were barely affected by the adjustment for preoperative platelet count, which was to fill in the missed values.

**Table 5:** Multivariate logistic regression analysis

Variables	OR (95%CI)	*P*
Education (Lower than middle school)	2.073 (0.545–7.888)	0.285
Gender (Female)	6.367 (0.728–55.689)	0.094
Duration of surgery (min)	1.009 (0.980–1.038)	0.556
Preoperative platelet (10^9/L)	1.014 (1.000–1.027)	0.043*

*OR* Odds ratio, *CI* Confidence interval

^*^P means *P* value < 0.05

In addition, the participants were divided into higher preoperative platelet group and lower preoperative platelet group by the cut-off value of 299. The characteristics and laboratory examinations were compared and listed in Table 6. We found that the occurrence of PND, percentage of females, preoperative platelet-to-lymphocyte ratio (PLR), and the level of postoperative platelet count were all increased, while the PT and INR were decreased in the higher preoperative platelet group.

**Table 6:** Difference between high preoperative platelet and low preoperative platelet groups divided by 229

Variables	High platelet group	Low platelet group	*P*
	> 229, *N* = 18	< 229, *N* = 52	
Age (y)	66(60.8–71)	67.5(63–75.5)	0.117
BMI (kg/m^2)^	25(21.6–27.2)	24.8(22.3–27.5)	0.783
PND			0.001**
Yes	9 (50%)	5 (9.6%)	
No	9 (50%)	47 (90.4%)	
Gender			0.039*
Male	2 (11.1%)	21 (40.4%)	
Female	16 (88.9%)	31 (59.6%)	
NYHA classification			0.145
≥ ΙΙ	3 (16.7%)	20 (38.5%)	
< ΙΙ	15 (83.3%)	32 (61.5%)	
Preoperative platelet (10^9/L)	252.5(245.3–276.0)	166.0(142.3–189.3)	< 0.001
Preoperative PLR	145.9(105.7–217.5)	113.8(80.7–153.4)	0.014*
Preoperative PT (s)	10.3(9.7–10.6)	10.6(10.2–10.9)	0.004**
Preoperative INR	0.94(0.90–0.97)	0.97(0.94–1.00)	0.041*
Postoperative platelet (10^9/L)	222.5(207.8–251.0)	150.5(132.5–178.3)	< 0.001

Data are presented as median with IQR for continuous variables and as the number for categorical variables. The *P*-value is calculated by the Mann-Whitney U test for continuous variables and by Fisher’s exact test for categorical variables

*PND* Perioperative neurocognitive disorders, *BMI* Body mass index, *NYHA* New York Heart Association, *PLR* Platelet-to-lymphocyte ratio, *PT* Prothrombin time, *INR* International normalized ratio

^*^P means *P*-value < 0.05

^**^P means P-value < 0.01

### Relationship between postoperative platelet count and occurrence of PND

The postoperative platelet in the early stage of PND was distinctly higher than that in the non-PND group (*P* = 0.003, Table 2). Moreover, as the ROC curve showed, the cut-off value (179.5, sen:0.857, spe:0.696) of postoperative platelet count was determined by Youden Index, and the AUC was 0.754 (*P* = 0.003, 95%CI 0.611–0.897, Fig. 2). The perioperative change of platelet and the change of PLR did not show a significant difference between PND and non-PND groups (Table 2).

Moreover, the participants were divided into higher postoperative platelet group and lower postoperative platelet group by the cut-off value of 179.5. The characteristics and laboratory examinations were compared and listed in Table 7. We found that the occurrence of PND, percentage of females, preoperative and postoperative platelet-to-lymphocyte ratio (PLR), and the level of preoperative platelet count were all significantly increased in the higher postoperative platelet group.

**Table 7:** Difference between high postoperative platelet and low postoperative platelet groups divided by 179.5

Variables	High platelet group	Low platelet group	*P*
	> 179.5, *N* = 29	< 179.5, *N* = 41	
Age (y)	66(61.5–71)	70(63–76)	0.08
BMI (kg/m^2^)	24.8(21.7–27.5)	25.2(22.5–27.2)	1.000
PND			< 0.001
Yes	12 (41.4%)	2 (4.9%)	
No	17 (58.6%)	39 (95.1%)	
Gender			0.005**
Male	4 (13.8%)	19 (46.3%)	
Female	25 (86.2%)	22 (53.7%)	
NYHA classification			1.000
≥ ΙΙ	10 (34.5%)	13 (31.7%)	
< ΙΙ	19 (63.5%)	28 (68.3%)	
Preoperative platelet (10^9/L)	235(203–258)	159(138–180.5)	< 0.001
Preoperative PLR	144.8(120.2–217.0)	98.6(80.0–139.0)	0.001**
Postoperative platelet (10^9/L)	212(196.5–237.0)	143(123.5–159.5)	< 0.001
Postoperative PLR	189.2(152.7–255.6)	149.1(105.6–193.7)	0.005**
Postoperative ALB (g/L)	38.3(37.0–40.3)	37.1(35.1–39.5)	0.048*

Data are presented as median with IQR for continuous variables and as number for categorical variables. The *P*-value is calculated by the Mann-Whitney U test for continuous variables and by Fisher’s exact test for categorical variables

*PND* Perioperative neurocognitive disorders, *BMI* Body mass index, *NYHA* New York Heart Association, *PLR* platelet-to-lymphocyte ratio, *ALB* Albumin

^*^P means *P*-value < 0.05

^**^P means P-value < 0.01

## Discussion

PND is a prevalent complication in aged people after major surgery, and it is hard to perform a scaled assessment due to patients’ poor condition and uncooperative behavior [32–34]. Thus, it is essential to find a new and easily accessible biomarker to predict PND occurrence at the early stage. This prospective observational study included 70 patients who took TKA or THA and analyzed the relationship between their perioperative platelet count and occurrence of PND on the third day after surgery. We found a significant increase in the perioperative platelet counts in the PND group than in the non-PND group. Besides, preoperative platelet count showed a significant difference in logistic analysis. It might be an independent risk factor and a potential predictor of PND. To our knowledge, this is the first study to estimate an association between perioperative platelet count in peripheral blood and the early stage of PND in patients undergoing major orthopedic surgery.

PND has been demonstrated to be associated with central neuroinflammation [13]. Surgery and anesthesia could stimulate peripheral inflammation, which could be manifested as increased IL-6, C reaction protein, and TNF-α [35–37]. Meanwhile, it was also reported that the peripheral inflammatory factors could go through the blood-brain barrier (BBB) more than usual because of the increased BBB permeability during pathological state [38]. Besides, the microglial cells in the aged brain are more sensitive to inflammatory factors and can secret more proinflammatory cytokines [39]. Once microglial cells are stimulated, central neuroinflammation occurs, which usually harms the hippocampus, resulting in the occurrence of PND [40]. In recent years, there were also increasing researches on platelet and suggested that platelet could exacerbate peripheral inflammation by self-degranulation or interacting with the immune cells through membrane surface receptors. For example, a bacterial infection could promote platelet to activate leukocytes via Toll-like receptors [41], and virus infection might lead to platelet releasing IL-1β, which is a core cytokine of the cascade of inflammation [42]. In addition, inflammatory mediators can make platelet self-degranulate and release chemokines, which can combine with receptors on the surface of immune cells [43]. And the previous study showed that higher platelet was associated with inflammation in colorectal cancer [44]. With more and more evidence, the platelet is deeply related to the severity of inflammation. Thus, in our study, a higher peripheral platelet level may be associated with more severe peripheral inflammation, which could cause more severe damage to the central nervous system and induce cognitive decline. And this might be the reason that preoperative platelet was an independent risk factor of the occurrence of PND at the early stage in our study. Compared with the stable condition before surgery, the patients undergoing the stress of surgery and anesthesia may suffer a higher risk of bacteria, virus infection, or non-infectious inflammation. Consistently, we found the patients in the PND group had higher postoperative platelet count. For the first time to explore the influence of platelet on the occurrence of PND after orthopedic surgery, more researches should be further performed to investigate the underlying pathological mechanism.

Tables 6 and 7 showed that patients with higher perioperative platelet count also had higher perioperative platelet-to-lymphocyte ratio (PLR). PLR has been proved to be a biomarker of inflammation in many diseases, which might explain why PLR is covaried with platelet [45–47]. However, the change of PLR showed no differences in the elderly patients between the two groups in our study. Therefore, more studies are required to ensure whether the change of PLR might be covariate with perioperative platelet. What’s more, lower PT and INR were observed in the higher preoperative platelet group. A past study has shown that high PT and INR and low platelet count could denote the same disease together [48]. So it was reasonable that lower PT and INR occurred in the higher preoperative platelet count group.

Eighty-four patients participated in this study, and 70 of them finished the whole process. We defined a decreased performance as the absolute value of reduced score more significant than 1.5 times SD of overall preoperative scores [31]. Then 14 of them developed PND, with a ratio of 14/70 (20%), which accorded with the reported percentage [7, 10, 49]. In terms of their baseline information, there was only a significant gender difference. Generally speaking, commonly reported risk factors are age and education [34]. As gender is often reported in cardiac surgery [50–52], there is limited evidence in orthopedic. Gender distribution in PND after major orthopedic surgery requires more studies. All of the patients were at a similar baseline preoperatively, while the patients in the PND group showed worse performance in several cognitive domains after surgery. On the one hand, surgery could induce inflammation and blood loss, which may cause cerebral hypoperfusion [53]. On the other hand, anesthesia may harm cognitive function by different drugs, methods, and depth [54–56]. In addition, the scores of PND patients were mainly decreased in word short-term recall, delayed recall, and delayed interference tests, indicating their neurocognitive damage were basically in aspects of short-term and delayed memory, almost consistent with that of the previous study [2]. Besides, we compared the baseline information and blood examinations between the mild PND group and severe PND group that no significant difference was displayed. The possible reason was that our mild and severe sample sizes were too small to gain a meaningful result. Larger samples are required to reduce the sampling bias.

As for the logistic multivariable regression and the prediction model, we could only take preoperative platelet count, gender, duration of surgery and education into consideration due to the size of the sample. Preoperative platelet count and gender were significantly different in our single variable analysis. While duration of surgery and education have been reported as comorbidities of PND in previous literature [57, 58]. With the result, we built a prediction model for PND occurrence, and its AUC of the ROC curve was 0.796. Because platelet count, gender, duration of surgery, and education were all easily accessible, our model might be meaningful to predict PND at the early stage after major orthopedic surgery. It can guide special care for the patients at high risk of PND in advance. Despite Dr. Wang and his colleges having built a prediction model for elderly orthopedic patients, the model was based on POD in 24 h [59]. Therefore, it is still meaningful to explore the role of preoperative platelet count and build a prediction model for patients in the early stage of PND after TKA and THA, while a larger scale of clinical investigation is also needed in the future.

Our study had several strengths. First, to our knowledge, we were the first to study the relationship between platelet count and occurrence of PND at the early stage after major orthopedic surgery in elderly patients, which could be helpful to patients’ care in the orthopedics department. Second, we provided a potential predictor of the early stage of PND. Preoperative platelet count can be examined much more quickly than a scale test. Third, we built a prediction model for the occurrence of PND, and it had an acceptable AUC. Forth, the data were credible because of well-trained investigators and reliable clinical examination in West China Hospital. On the contrary, our main limitation was the relatively small sample size, which could cause some bias and limit our model. Generally speaking, the multivariable logistic regression analyses should be used with a minimum of 10 events per predictor variable. So we can only take preoperative platelet count, education, gender and duration of surgery into consideration. Moreover, the patients were usually discharged from the hospital in 3 days, making it difficult for us to collect blood samples in a more extended follow-up period. Last but not least, although platelet counts in PND patients were elevated, they were still within the normal range. However, the elevated platelet could still give us clues to monitor and intervene in the elderly patients’ cognitive function at the early stage.

In summary, our prospective observational study has demonstrated that a higher perioperative platelet count in peripheral blood might be the biomarkers of PND at the early stage in aged patients after major orthopedic surgery. And preoperative platelet count could be a potential biomarker of the early stage of PND. Thus, elderly patients in the orthopedics department with preoperative platelet higher than 229 or with a high probability of PND in our model should be taken special care of after surgery. Besides, memory and cognitive function should be specially trained perioperatively to keep an excellent cognitive condition.

## Conclusion

Perioperative higher levels of platelet count were associated with a higher risk of occurrence of PND at the early stage in patients after major orthopedic surgery. Therefore, patients with a higher perioperative platelet count should be treated more carefully in many aspects to decrease PND occurrence. Moreover, preoperative platelet count seems to be a clinically valuable biomarker to predict PND early after major orthopedic surgery. In contrast, the platelet count is easily accessible and always contained by the blood routine examinations. And a prediction model constructed including education, gender, duration of surgery, and preoperative platelet count would be effective in predicting the occurrence of PND. However, more extensive clinical investigations and basic experiment studies are required to ensure our findings and further clarify the potential mechanisms and communications.

## Supplementary Information

**Additional file 1: Supplementary Table 1.** Preoperative variables of mild and severe PND groups.

**Additional file 2: Supplementary Table 2.** Postoperative variables of mild and severe PND groups.

## Data Availability

The datasets used and/or analyzed during the current study are available from the corresponding author on reasonable request.

## Abbreviations

POCD: Postoperative cognitive dysfunction
POD: Postoperative delirium
PND: Perioperative neurocognitive disorders
TKA: Total knee arthroplasty
THA: Total hip arthroplasty
MMSE: Mini Mental Stare Examination
BMI: Body mass index
SD: Standard deviation
IQR: Interquartile range
ROC: Receiver operating curve
AUC: Area under the curve
OR: Odds ratio
PLR: Platelet-to-lymphocyte ratio
BBB: Blood brain barrier
