# Supplementary material for: Perioperative platelet count in peripheral blood is associated with the early stage of PND after major orthopedic surgery: a prospective observational study
Source: BMC Geriatr. 2022 Mar 14;22:200. doi: 10.1186/s12877-022-02899-7 (PMC8919528; doi:10.1186/s12877-022-02899-7)
Supplement: Supplementary file 1 — Additional file 1: Supplementary Table 1. Preoperative variables of mild and severe PND groups. [file 12877_2022_2899_MOESM1_ESM.docx]

**Supplementary Table 1** **Preoperative variables of mild and severe PND groups**

| Preoperative variables | Mild PND group | Severe PND group | *P* |
| --- | --- | --- | --- |
|  | N=7 | N=7 |  |
| Age (y) | 68（66-71） | 72（61-75） | 1.000 |
| BMI (kg/m^2)^ | 26.9（21.6-29.1） | 24.8（21.7-25.8） | 0.805 |
| Gender |  | | 1.000 |
| Male | 1 (14.3%) | 0 (0%) |  |
| Female | 6 (85.7%) | 7 (100%) |  |
| Education |  | | 1.000 |
| No lower than middle school | 4 (57.1%) | 4 (57.1%) |  |
| Lower than middle school | 3 (42.9%) | 3 (42.9%) |  |
| Operation type |  | | 1.000 |
| TKA | 2 (28.6%) | 3 (42.9%) |  |
| THA | 5 (71.4%) | 4 (57.1%) |  |
| NYHA classification |  | | 0.559 |
| ≥Ⅱ | 3 (42.9%) | 1 (14.3%) |  |
| <Ⅱ | 4 (57.1%) | 6 (85.7%) |  |
| Hypertension |  | | 0.192 |
| Yes | 0 (0%) | 3 (42.9%) |  |
| No | 7 (100%) | 4 (57.1%) |  |
| If atherosclerosis in lower limbs |  | | 0.592 |
| Yes | 4 (57.1%) | 2 (28.6%) |  |
| No | 3 (42.9%) | 5 (71.4%) |  |
| [Hemoglobin](#/javascript:;) (g/L) | 138（121-143） | 135（126-150） | 1.000 |
| [Hematokrit](C:/Users/wrq/AppData/Local/youdao/dict/Application/8.9.8.0/resultui/html/index.html#/javascript:;) | 0.43（0.36-0.44） | 0.41（0.38-0.46） | 0.902 |
| Platelet (10^9/L) | 235（192-275） | 243（130-252） | 0.620 |
| Leukocyte (10^9/L) | 5.87（4.74-7.65） | 5.82（4.64-6.53） | 1.000 |
| Neutrophil percentage (%) | 61.7（55.3-67.1） | 60.1（47.6-74.0） | 1.000 |
| Neutrophil count (10^9/L) | 3.85（3.05-4.23） | 3.59（3.10-4.32） | 1.000 |
| Lymphocyte (10^9/L) | 1.87（1.35-2.31） | 1.52（0.79-1.92） | 0.535 |
| PLR | 142.2（101.7-167.4） | 130.9（70.4-246.8） | 1.000 |
| Monocyte (10^9/L) | 0.37（0.35-0.49） | 0.37（0.35-0.50） | 0.902 |
| ALT (U/L) | 15（12-21） | 17（14-38） | 0.535 |
| AST (U/L) | 21（18-30） | 20（19-30） | 0.902 |
| ALB (g/L) | 44.6（38.5-45.6） | 43.8（41.6-47.7） | 0.383 |
| TBil (μmol/L) | 11.9（10-13.1） | 9.4（7.4-17.1） | 0.620 |
| HDL (mmol/L) | 1.61（1.13-1.83） | 1.44（1.22-1.5） | 0.620 |
| LDL (mmol/L) | 2.98（2.31-3.85） | 3.99（2.63-4.38） | 0.318 |
| Serum creatinine (μmol/L) | 61（58-71） | 66（54-74） | 0.902 |
| Blood glucose (mmol/L) | 5.9（5.1-6.2） | 5.3（4.8-5.7） | 0.383 |
| PT (s) | 10.5（9.2-11.2） | 10.3（9.9-10.7） | 0.535 |
| APTT (s) | 25.8（24.9-26.6） | 25.4（24.1-27.1） | 0.805 |
| INR | 0.97（0.91-1.01） | 0.97（0.91-0.97） | 0.620 |
| Duration of surgery (min) | 61（47-90） | 62（55-113） | 0.710 |
| Duration of anesthesia (min) | 109（100-146） | 121（99-155） | 0.620 |

Data are presented as median with IQR for continuous variables and as number for categorical variables. The P-value is calculated by the Mann-Whitney U test for continuous variables and by Fisher’s exact test for categorical variables. P* means P-value < 0.05. P** means P-value < 0.01. We diagnose the systolic pressure ≥140 mmHg and/or diastolic pressure ≥90 mmHg preoperative as hypertension. PND, perioperative neurocognitive disorders; BMI, body mass index; TKA, total knee arthroplasty ;THA, total hip arthroplasty; NYHA, New York Heart Association; PLR, platelet-to-lymphocyte ratio; ALT, Alanine aminotransferase; AST, Aspartate aminotransferase; ALB, albumin; TBil, [total](C:/Users/wrq/AppData/Local/youdao/dict/Application/8.9.8.0/resultui/html/index.html#/javascript:;) [bilirubin](C:/Users/wrq/AppData/Local/youdao/dict/Application/8.9.8.0/resultui/html/index.html#/javascript:;); HDL, [high](C:/Users/wrq/AppData/Local/youdao/dict/Application/8.9.8.0/resultui/html/index.html#/javascript:;) [density](C:/Users/wrq/AppData/Local/youdao/dict/Application/8.9.8.0/resultui/html/index.html#/javascript:;) [lipoprotein](C:/Users/wrq/AppData/Local/youdao/dict/Application/8.9.8.0/resultui/html/index.html#/javascript:;); LDL, low [density](C:/Users/wrq/AppData/Local/youdao/dict/Application/8.9.8.0/resultui/html/index.html#/javascript:;) [lipoprotein](C:/Users/wrq/AppData/Local/youdao/dict/Application/8.9.8.0/resultui/html/index.html#/javascript:;); PT, [prothrombin](C:/Users/wrq/AppData/Local/youdao/dict/Application/8.9.8.0/resultui/html/index.html#/javascript:;) [time](C:/Users/wrq/AppData/Local/youdao/dict/Application/8.9.8.0/resultui/html/index.html#/javascript:;); APTT, [activated](C:/Users/wrq/AppData/Local/youdao/dict/Application/8.9.8.0/resultui/html/index.html#/javascript:;) [partial](C:/Users/wrq/AppData/Local/youdao/dict/Application/8.9.8.0/resultui/html/index.html#/javascript:;) [thromboplastin](C:/Users/wrq/AppData/Local/youdao/dict/Application/8.9.8.0/resultui/html/index.html#/javascript:;) [time](C:/Users/wrq/AppData/Local/youdao/dict/Application/8.9.8.0/resultui/html/index.html#/javascript:;); INR, [international](C:/Users/wrq/AppData/Local/youdao/dict/Application/8.9.8.0/resultui/html/index.html#/javascript:;) [normalized](C:/Users/wrq/AppData/Local/youdao/dict/Application/8.9.8.0/resultui/html/index.html#/javascript:;) [ratio](C:/Users/wrq/AppData/Local/youdao/dict/Application/8.9.8.0/resultui/html/index.html#/javascript:;).

**Supplementary file 1: Word.doc. Preoperative variables of mild and severe PND groups.** It compared the preoperative information between mild PND group and severe PND group.
