# Supplementary material for: Perioperative platelet count in peripheral blood is associated with the early stage of PND after major orthopedic surgery: a prospective observational study
Source: BMC Geriatr. 2022 Mar 14;22:200. doi: 10.1186/s12877-022-02899-7 (PMC8919528; doi:10.1186/s12877-022-02899-7)
Supplement: Supplementary file 2 — Additional file 2: Supplementary Table 2. Postoperative variables of mild and severe PND groups. [file 12877_2022_2899_MOESM2_ESM.docx]

**Supplementary Table 2** **Postoperative variables of mild and severe PND groups**

| Postoperative variables | Mild PND group | Severe PND group | *P* |
| --- | --- | --- | --- |
|  | N=7 | N=7 |  |
| [Hemoglobin](#/javascript:;) | 119（117-132） | 118（108-125） | 0.535 |
| [Hematokrit](C:/Users/wrq/AppData/Local/youdao/dict/Application/8.9.8.0/resultui/html/index.html#/javascript:;) | 0.36（0.34-0.41） | 0.35（0.33-0.41） | 0.805 |
| Platelet (10^9/L) | 208（180-254） | 208（190-219） | 0.620 |
| Leukocyte (10^9/L) | 10.2（9.89-15.42） | 11.82（7.64-13.27） | 0.902 |
| Neutrophil percentage (%) | 85.7（84.0-88.7） | 83.6（82.3-86.9） | 0.259 |
| Neutrophil count (10^9/L) | 9.08（8.48-13.21） | 9.82（6.77-11.12） | 0.902 |
| Lymphocyte (10^9/L) | 1.31（0.78-1.41） | 1.11（0.75-1.57） | 0.805 |
| PLR | 189.1（158.8-230.8） | 171.2（117.1-293.3） | 0.805 |
| Change of PLR | 75.5（33.1-91.0） | 9.2（-9.5-85.6） | 0.259 |
| Monocyte (10^9/L) | 0.73（0.33-0.81） | 0.59（0.27-0.78） | 0.620 |
| ALT (U/L) | 18（15-28） | 15（14-31） | 0.710 |
| AST (U/L) | 32（21-45） | 21（19-34） | 0.383 |
| ALB (g/L) | 37.3（36.2-42.2） | 36.6（35.7-40.1） | 0.620 |
| TBil (μmol/L) | 11.3（8.9-18.6） | 11（8.9-11.8） | 0.620 |
| HDL (mmol/L) | 1.6（1.5-1.7） | 1.3（1.2-1.5） | 0.097 |
| LDL (mmol/L) | 2.5（2.2-3.5） | 3.3（2.0-3.6） | 0.805 |
| Serum creatinine (μmol/L) | 63（60-67） | 63（54-80） | 0.902 |
| Blood glucose (mmol/L) | 7.3（6.4-8.2） | 7.0（5.5-7.1） | 0.165 |

Data are presented as median with IQR for continuous variables and as number for categorical variables. The P-value is calculated by the Mann-Whitney U test for continuous variables and by Fisher’s exact test for categorical variables. P* means P-value < 0.05. P** means P-value < 0.01. PND, perioperative neurocognitive disorders; PLR, platelet-to-lymphocyte ratio; ALT, Alanine aminotransferase; AST, Aspartate aminotransferase; ALB, albumin; TBil, [total](C:/Users/wrq/AppData/Local/youdao/dict/Application/8.9.8.0/resultui/html/index.html#/javascript:;) [bilirubin](C:/Users/wrq/AppData/Local/youdao/dict/Application/8.9.8.0/resultui/html/index.html#/javascript:;); HDL, [high](C:/Users/wrq/AppData/Local/youdao/dict/Application/8.9.8.0/resultui/html/index.html#/javascript:;) [density](C:/Users/wrq/AppData/Local/youdao/dict/Application/8.9.8.0/resultui/html/index.html#/javascript:;) [lipoprotein](C:/Users/wrq/AppData/Local/youdao/dict/Application/8.9.8.0/resultui/html/index.html#/javascript:;); LDL, low [density](C:/Users/wrq/AppData/Local/youdao/dict/Application/8.9.8.0/resultui/html/index.html#/javascript:;) [lipoprotein](C:/Users/wrq/AppData/Local/youdao/dict/Application/8.9.8.0/resultui/html/index.html#/javascript:;).

**Supplementary file 2: Word.doc. Postoperative variables of mild and severe PND groups.** It compared the postoperative information between mild PND group and severe PND group.
